# External Stimulus in Cholera

**Published:** 1854-02

**Authors:** 


External Stimulus in Cholera.—The resources of professional judgment in the treatment of Cholera are not so numerous and efficient as to render a somewhat novel remedy unacceptable to those whose lot it may be to undertake the charge of many cases. I am indebted to a lady of heroic mind and great intelligence for the hints which led me to adopt in the collapse of cholera the powerful external stimulus which I shall presently describe. It appears that in the most desperate cases, even when life has appeared extinct, the native Indians are accustomed to apply the actual cautery freely to the abdomen, not unfrequently with the happy result of restored vitality. The remedy, based on the same principle, which I have employed in three cases with complete success in the borough jail of Newcastle, is precisely similar in kind, though somewhat less harsh and formidable in degree. A

piece of linen dipped in brandy is placed over the epigastrium or abdomen, and ignited; the brandy burns away in a minute or so, producing a considerable feeling of pain, which renders it necessary to secure the hands of the patient. Slight vesication will probably follow, and, if successful, in a short time heart and pulse begin to return, and the feelings of the patient are greatly improved; vomiting will also generally be put a stop to. It is probable that the application may soon require repetition, the situation being somewhat varied. In one case, now convalescent, in which death was apparently close at hand, the most complete effect was produced by a third application along the spine in the lumbar region. Time will not permit me to give details of cases, but having now tried the "brandy blister" in seven or eight cases of total collapse, I repeat that in three it has been entirely successful, and in others has had the effect of temporarily rousing the patients; and if, as experience has taught me, in some of these it had been repeated with more energy, greater success might possibly have resulted. The patients now convalescent say that it is a severe remedy, but are quite conscious of its beneficial effects, and attribute to its use, without any hesitation, their restoration from impending death.—*Mr. Greenhow in London Lancet.*

